# A Comparative Study on the Wake Sensing Mechanism of a Seal Whisker-Shaped Cylinder

**DOI:** 10.3390/s25113529

**Published:** 2025-06-03

**Authors:** Yitian Mao, Hao Chang, Yaohong Wang, Dekui Yuan, Yingxue Lv, Ziyu Song

**Affiliations:** 1 Department of Mechanics, School of Mechanical Engineering, Tianjin University, Tianjin 300072, China; maoyitian@tju.edu.cn; 2 Department of Port Engineering, School of Civil Engineering, Tianjin University, Tianjin 300072, China; changhao_tju@foxmail.com (H.C.); 2023205351@tju.edu.cn (Z.S.); 3 School of Mathematics, Tianjin University, Tianjin 300072, China; 4 State Key Laboratory of Hydraulic Engineering Intelligent Construction and Operation, Tianjin University, Tianjin 300072, China; 5 CCCC First Harbor Engineering Company Ltd. (Key Laboratory of Coastal Engineering Hydrodynamics, CCCC), Tianjin 300461, China; lvyingxue@ccccltd.cn

**Keywords:** seal whisker sensor, particle image velocimetry, spectral proper orthogonal decomposition, lift signal

## Abstract

This study conducts water channel experiments to measure and compare the lift forces experienced by the seal whisker-shaped sensor and the circular cylinder sensor in both no vortex generator and the wake of two types of circular cylinders. Particle Image Velocimetry (PIV) is employed to capture the velocity fields of the cylinder wake and the surrounding flow of the seal whisker-shaped cylinders. Spectral analysis of the lift signals reveals that both cylinder types exhibit a primary peak close to the vortex shedding frequency of the upstream circular cylinder. However, seal whisker-shaped cylinders demonstrate relatively weaker components in their lift signals that do not align with the primary frequency, indicating a stronger sensing capability of the upstream cylinder’s wake. Modal analysis using Spectral Proper Orthogonal Decomposition (SPOD) on the PIV-measured velocity fields shows that the lift signals of both cylinder types are primarily induced by the vortices in the upstream cylinder’s wake.

## 1. Introduction

Inspired by the ability of seals to perceive their surroundings by sensing water flow disturbances through their whiskers, researchers have begun investigating their applications in biomimetic sensors. For example, Eberhardt et al. [1] designed a fluid motion sensor array that emulates the whiskers of harp seals, enabling the detection and tracking of underwater wakes. This sensor, known as the Wake Information Detection and Tracking System, features multiple whisker-like elements that respond sensitively to hydrodynamic disturbances encountered during underwater movement. Liu et al. [2] designed and fabricated a piezoresistive seal whisker sensor and validated its capability to sense oscillatory incoming flow and vortices in the flow through water channel experiments. More recently, Zheng et al. [3,4] developed a cantilever graphene sensor inspired by seal whiskers and conducted wake measurement experiments, further advancing research in this field.

To obtain a deeper understanding of the tracking capabilities of seal whisker sensors, numerous researchers have conducted in-depth investigations into their flow field characteristics. Some studies have focused on the mechanisms by which seal whiskers suppress vortex-induced vibrations (VIV) in uniform inflows. Schulte-Pelkum et al. [5] discovered that seal whiskers exhibit significant vibrations in the wake of fish, but effectively suppress vortex-induced vibrations in high-speed uniform flows. Hanke et al. [6] attributed this VIV suppression to the unique undulated, asymmetric, and elliptical shape of seal whiskers. The suppression of VIV not only reduces noise generated by vortex-induced vibrations but also significantly enhances the signal-to-noise ratio, endowing seal whiskers with superior detection capabilities. They also employed Particle Image Velocimetry (PIV) experiments at Re = 500 and Direct Numerical Simulation (DNS) to reveal that the wake behind seal whiskers exhibits a complex fully 3D structure with extended vortex formation lengths. Bunjevac [7] used PIV to measure the wake fields of smooth and undulated whiskers, demonstrating that the power spectral density of the wake behind undulated whiskers is 40% lower than that of smooth whiskers. Lyons et al. [8] investigated the influence of seal whisker geometric parameters on their fluid dynamic correlations, finding that higher undulation offsets significantly reduce the mean drag coefficient and the fluctuating lift coefficient. Chu et al. [9] employed a Large Eddy Simulation (LES) model to simulate the flow field around seal whiskers at a Reynolds number of 20,000 and studied the impact of turbulence on the lift and drag forces experienced by whisker-like cylinders. Zheng [10] conducted numerical simulations to examine the noise generated by nine different seal whisker-like cylinder shapes under the influence of VIV, aiming to design improved biomimetic sensor models. More recently, Dunt [11] studied the effect of different wavelength parameters on the vortex shedding frequency of seal whiskers.

Other researchers have focused on the forces and responses experienced by seal whiskers in vortical inflows. Beem [12] conducted experiments which revealed that seal whisker models can vibrate at frequencies close to the vortex shedding frequency of a disturbance source, even when the distance to the source exceeds 17 times the whisker model’s diameter. In contrast, circular cylinders exhibited vibration frequencies that deviated from the disturbance source’s vortex shedding frequency. Through dye visualization, it was observed that whisker models placed in the wake of a circular cylinder exhibited a slaloming vibration path between the cylinder’s wake vortices. This phenomenon causes the vibration frequency of seal whiskers to lock onto the vortex shedding frequency of the upstream cylinder. Dulac [13] compared the amplitudes and frequencies of vortex-induced vibrations of seal whiskers at angles of attack of 0° and 90° in the wake of a circular cylinder, demonstrating the complexity of the wake flow behind the whiskers at a 90° angle of attack. Zheng [4] measured the lateral vibration frequency of a seal whisker sensor in the presence of an upstream cylinder acting as a vortex source, finding that the vibration frequency closely approximated the vortex shedding frequency in the cylinder’s wake. Additionally, a comparison between the seal whisker-shaped cylinder and circular cylinders as sensors revealed that, while circular cylinders experience stronger vortex-induced vibrations in uniform flows compared to wake-induced vibrations, seal whisker models exhibit stronger wake-induced vibrations than uniform flow-induced vibrations. Zhao [14] compared the vibration and force characteristics of a seal whisker model at different angles of attack in the wake of an oscillating fish tail.

In the analysis of flow fields behind various bluff bodies, Proper Orthogonal Decomposition (POD) and Dynamic Mode Decomposition (DMD) are two widely applied modal decomposition methods. In 2012, Witte et al. [15] discovered the mechanism by which seal whiskers attenuate flow-induced forces. Through POD analysis of pressure, they found that the pressure on the upper half of the seal whisker is in the opposite direction to that on the lower half, leading to mutual cancelation and thereby reducing the lift force experienced by the whisker. Wang and Liu [16] employed DMD to analyze the vortex dynamics of a harp seal whisker model at Re = 1800, using Particle Image Velocimetry (PIV) measurements, and compared it with cylinders, elliptical cylinders, and wavy cylinders of equivalent hydraulic diameter. The results showed that the harp seal whisker model exhibited a significantly reduced and highly stable recirculation zone, with notably lower streamwise and spanwise velocity fluctuations. The different vortex shedding frequencies on the dorsal and ventral surfaces of the whisker, along with the complex and unsteady vortex shedding process, effectively suppressed vortex-induced vibrations. However, traditional POD ensures spatial orthogonality but not temporal orthogonality, while DMD ensures temporal orthogonality but not spatial orthogonality. To address this, Towne et al. [17] proposed the Spectral Proper Orthogonal Decomposition (SPOD) method, which uses a space-time inner product to ensure both spatial and temporal orthogonality. In 2020, Zhang et al. [18] employed SPOD to analyze the three-dimensional wake field of a rectangular cylinder. Li et al. [19] used SPOD to analyze the three-dimensional wake field of a train.

While significant progress has been made in understanding how seal whiskers attenuate flow-induced forces in uniform flow fields, and existing work has established their ability to capture disturbance signals, the specific fluid dynamic mechanisms that enable them to effectively capture these signals while concurrently reducing noise in flows with vortical perturbations remain less comprehensively explored. To provide theoretical guidance for optimizing the flow signal capture capabilities of seal whisker-like sensors, this study focuses on the flow field structures upstream and downstream of the seal whisker-shaped cylinder and compares them with those of circular cylinders with the same equivalent diameter. Using Particle Image Velocimetry (PIV) technology, we measure the flow fields upstream and downstream of seal whisker-shaped cylinder and comparison cylinders in vortical inflows. We then employ the Spectral Proper Orthogonal Decomposition (SPOD) method to analyze the coherent structures in the flow field around the seal whisker-shaped cylinder in disturbed inflows, aiming to uncover the mechanisms by which the seal whisker-shaped cylinder captures disturbance signals. According to our previous study [20], obstacle identification using a seal whisker sensor with a convolutional neural network (CNN) primarily depends on the spectral characteristics of the lift signal. When sensors are arranged to form a sensor array, it is common to have multiple sensors placed in a staggered configuration, where downstream sensors are located within the wake flow generated by the upstream sensors. In this study, we measured the lift signals produced by both the seal whisker sensor and the cylindrical sensor in the wake of a vortex generator, and we analyzed these signals in combination with their corresponding flow field characteristics. The results offer meaningful insights that can inform the design and optimization of underwater detection systems and sensor arrays.

The structure of this paper is as follows: Section 2 describes the experimental methods and the SPOD algorithm in detail. Section 3 presents the PIV measurements of the flow fields around seal whisker-shaped cylinder and circular cylinders in vortical inflows, as well as the forces experienced by these two cylinders’ types in such flows. Section 4 provides an in-depth analysis and discussion of the results and mechanisms. Finally, the conclusions summarize the main findings of this research.

## 2. Experimental Setup

The experiment was conducted in a low-turbulence circulating water channel located at the Fluid Mechanics Laboratory of Tianjin University. The test section of the water channel has dimensions of 2370 mm in length, 400 mm in height, and 306 mm in width. The side walls and bottom wall of the channel are made of glass to facilitate PIV imaging. The top of the water channel is open, with a free water surface, and the water depth during experiments was maintained at 330 mm. The water flow in the channel is driven by an electric motor, with flow velocities ranging from 0 to 0.4 m/s, and the turbulence intensity was less than 1% at a flow velocity of 0.4 m/s. To ensure the uniformity of the incoming flow, a settling chamber, a honeycomb, six screens, and a contraction section were placed in sequence upstream of the test section. All experiments were performed under the condition of an incoming flow velocity of U=0.15 m/s in the test section.

**Figure 1:**
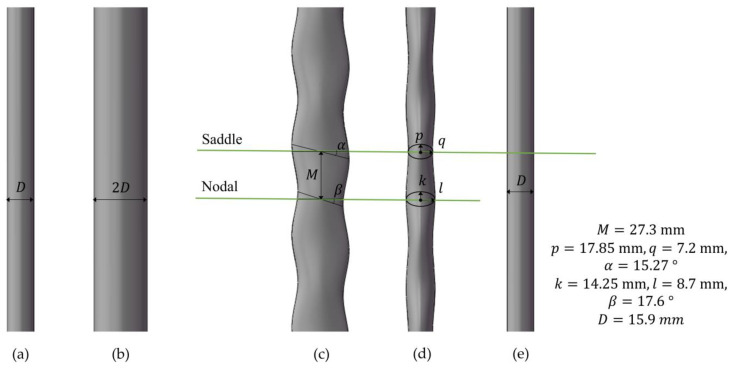
Schematic diagram of vortex generation cylinders for type C1 (**a**) and type C2 (**b**); seal whisker-shaped cylinder (seal whisker sensor) in (**c**) from side view direction; (**d**) from flow-facing direction; (**e**) side view of cylindrical sensor; and two measurement planes (Saddle plane and Nodal plane) in (**c**–**e**).

To ensure the comparability and generalizability of the experimental results, we adopted the seal whisker parameters proposed by Hanke et al. [6]. Based on these parameters, we fabricated a 30-times scaled-up seal whisker model, as shown in Figure 1c,d. The seal whisker-style cylinder was made of resin, and no significant displacement or deformation was observed during the experiments. The shape parameters of this seal whisker-style cylinder included the lengths of the major and minor axes of two controlling elliptical cross-sections (p, q, k, l), the distance between the two controlling elliptical cross-sections (M), and the inclination angle of the two controlling elliptical cross-sections (α, β). The parameters of the spotted seal whisker-style cylinder used in the experiment were M=27.3 mm, p=17.85 mm, q=7.2 mm, α=15.27 °, k=14.25 mm, l=8.7 mm, β=17.6 °. The narrow-face equivalent diameter of the seal whisker-style cylinder in the Y-Z plane was

(1) $$D=2\times \frac{q+l}{2}=15.90\;\mathrm{mm}$$

The wide-face equivalent diameter of the seal whisker-style cylinder in the Y-Z plane was

(2) $$D_{\mathrm{wide}}=2\times \frac{p\cos\alpha +k\cos\beta }{2}=30.81\;\mathrm{mm}.$$

The length of the cylinder is 343 mm.

For comparison, a cylindrical probe with the same diameter and length as the seal whisker was also utilized in this experiment. Two cylinders of different diameters, denoted as C1 and C2 (see Figure 1a,b), were used as vortex generation sources. All probes and vortex generation sources were immersed to a length of 273 mm, corresponding to five wavelengths of the seal whisker. At a flow velocity of 0.15 m/s, the Reynolds number is 2385 when calculated using the characteristic length of the seal whisker sensor, the cylindrical sensor, or the type C1 cylinder. For the type C2 cylinder, the Reynolds number is 4770 based on its characteristic length.

**Figure 2:**
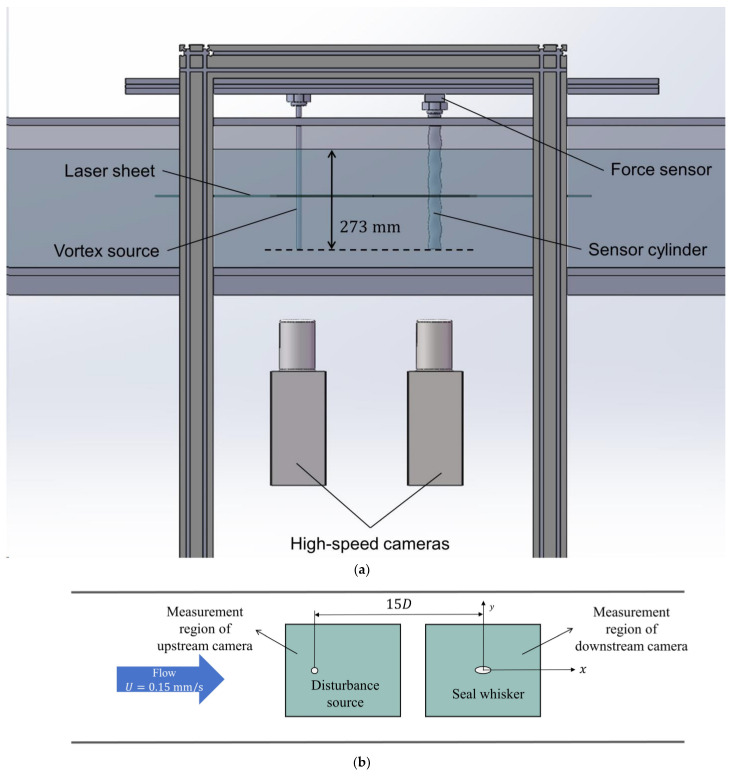
Schematic of PIV experimental setup ((**a**) side view, (**b**) bottom view).

The experimental setup is illustrated in Figure 2. The vortex generation sources and the probes were mounted on supports fixed to the water channel and aligned along the channel’s centerline, with the vortex generation sources positioned upstream of the probes. Both were oriented vertically downward with free bottom ends, positioned 57 mm above the channel floor. A force sensor was installed at the top fixed support of the probe to measure the lift force experienced during the experiment.

The illumination equipment used in the experiment was the Vlite-Hi-527 laser (manufactured by Beamtech Optronics Co., Ltd. (Beijing, China)), with an emission wavelength of 527 nm, a maximum pulse frequency of 10 kHz, and a single pulse energy of 15.5 mJ. Two high-speed cameras (X150-M, manufactured by HF AGILE DEVICE Co., Ltd. (Hefei, China)) were employed synchronously (ASG8100, manufactured by CIQTEK Co. (Hefei, China)) to capture particle images downstream of the disturbance source and around the sensor. The field of view for the camera downstream of the disturbance source was in the range of −16D<x<−7D and −3D<x<3D, while the field of view around the receiver was in the range of −5D<x<5D and −3D<x<3D. Hollow glass microspheres (density 1.1 g/mL, mean diameter 5 μm, manufactured by Beiting Measurement Technique Co., Ltd. (Beijing, China)) were seeded into the water channel as tracer particles. The frame rate of the two cameras was set to 40 Hz during the experiments.

The flow field around the seal whisker sensor was imaged in two horizontal planes, as shown in Figure 1c,d, while the flow field around the cylindrical sensor was imaged only in the plane in Figure 1e. Each experimental condition was recorded for a duration of 120 s, yielding 4800 pairs of flow field particle images. The velocity field was obtained through particle image analysis using PIVlab [21]. These particle images were processed using the multi-grid cross-correlation algorithm to obtain 4800 snapshots of the velocity field at different time instants. These particle images were processed using the multi-grid cross-correlation algorithm to obtain 4800 snapshots of the velocity field at different time instants. During multi-grid cross-correlation processing, the final interrogation window size was 16 × 16 pixels, with an overlap rate of 50% in both horizontal and vertical directions. Consequently, on the saddle plane, the resolution of the velocity field is 0.74 mm downstream of the vortex source and 0.53 mm in the vicinity of the sensor. Similarly, on the nodal plane, the velocity field resolution is 0.79 mm downstream of the vortex source and 0.56 mm around the sensor.

Additionally, separate PIV measurements were conducted for the flow field with each vortex generation source and each vortex sensor individually, serving as a baseline for comparing flow field changes. Furthermore, a force sensor was used to measure the lift force experienced by the sensor at a sampling frequency of 1 kHz for a continuous duration of 120 s for each experimental condition.

## 3. Results

### 3.1. The Spectra of Lift Force Signals

**Figure 3:**
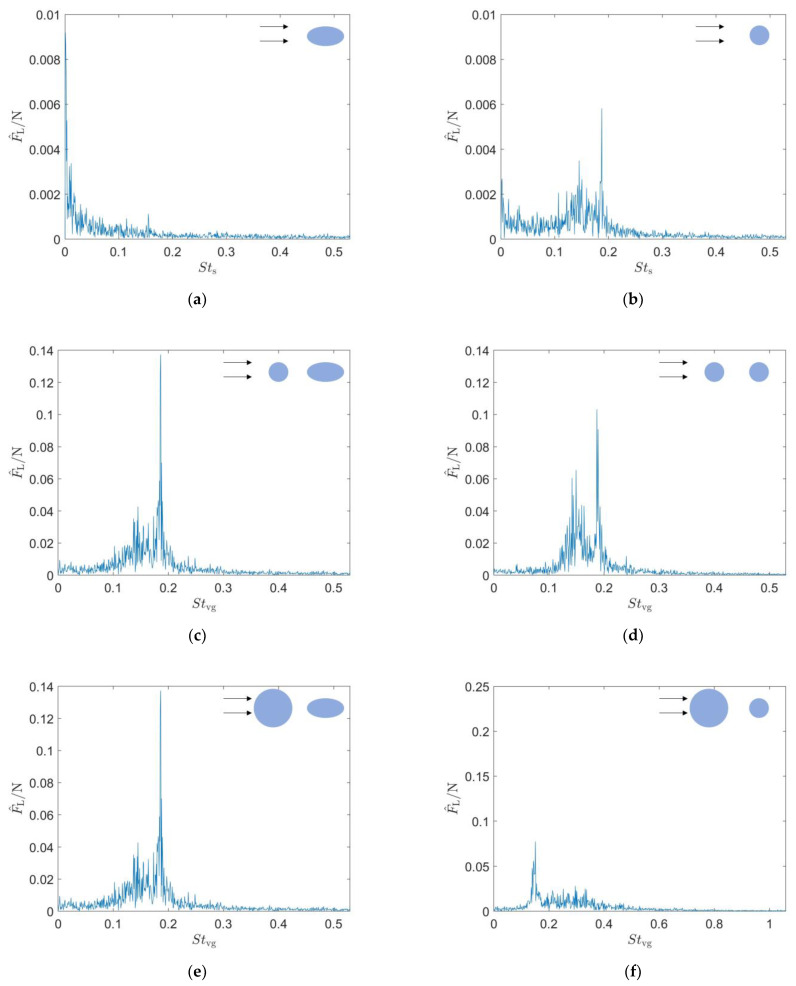
Spectra of the lift force signals for the sensors in three different incoming flows (**a**,**b**): no vortex generator. (**c**,**d**) In the wake of type C1 cylinder. (**e**,**f**) In the wake of type C2 cylinder. The left column shows the spectra for the seal whisker sensor, and the right column shows the spectra for the circular cylinder sensor.

First, the lift signals measured from the seal whisker sensor and the cylindrical sensor under different experimental conditions were subjected to fast Fourier transform (FFT). Figure 3 illustrates the FFT spectra of the lift forces ${\hat{F}}_{L}$ experienced by the two sensors (the seal whisker sensor and the circular cylinder sensor), under three different experimental conditions. Strouhal is defined as St=fl/v, where f denotes the vortex shedding frequency, l represents the characteristic length, and v corresponds to the free-stream velocity. Since the experiment involves two objects—the vortex generator and the sensor—we calculate the Strouhal number $St_{\mathrm{vg}}$ using the characteristic length of the vortex generator when it is present, and the Strouhal number $St_{s}$ using the characteristic length of the sensor when the vortex generator is absent. Table 1 shows peak values derived from FFT spectra of the lift forces. When no vortex generator is present, the lift force signal of the cylindrical sensor exhibits a distinct peak at $St_{s}=0.188$ with an amplitude of approximately 0.006 N. This indicates that when no vortex generator is present, the cylindrical sensor introduces noise into the lift force signal, while the seal whisker sensor does not.

When the C1 type cylindrical vortex generation source is present upstream, the lift force signal of the cylindrical sensor exhibits two peaks, with the higher peak at $St_{\mathrm{vg}}=0.187$ and an amplitude of 0.103 N, and the lower peak at $St_{\mathrm{vg}}=0.149$ and an amplitude of 0.065 N. The lift force spectrum of the seal whisker sensor also shows two peaks, with the higher peak at $St_{\mathrm{vg}}=0.186$ and an amplitude of 0.137 N, and the lower peak at a frequency of $St_{\mathrm{vg}}=0.145$ and an amplitude of 0.043 N. The secondary peak of the seal whisker sensor is less intense than that of the cylindrical sensor, indicating that the seal whisker sensor has better noise suppression characteristics when the upstream vortex generation source is the C1 type cylinder.

When the C2 type cylindrical vortex generation source is present upstream, both sensors exhibit a prominent peak in their lift force signals. The cylindrical sensor experiences a peak at $St_{\mathrm{vg}}=0.151$ with an amplitude of 0.077 N, along with a series of higher-amplitude components distributed between $St_{\mathrm{vg}}=0.191$ and $St_{\mathrm{vg}}=0.360$. The seal whisker sensor’s lift force spectrum shows a pronounced peak at $St_{\mathrm{vg}}=0.161$ with an amplitude of 0.214 N. Under the C2 type cylindrical vortex generator condition, the highest peak in the lift force signal of the seal whisker sensor is three times that of the cylindrical sensor, indicating that the seal whisker sensor receives a much stronger lift force signal than the cylindrical sensor.

### 3.2. SPOD Analysis

To obtain a deeper understanding of the spatiotemporal characteristics of the flow field, we employed the SPOD method to perform modal decomposition on the flow field data obtained from PIV. Unlike the POD algorithm, the modes extracted by SPOD contain information at a single frequency and are orthogonal with respect to the space-time inner product. The SPOD analysis of the experimental results was performed using Schmidt’s algorithm [17]. The modal energy of SPOD was nondimensionalized with $U^{2}$ as the reference during the calculation, plotting, and analysis, where U=0.15 m/s.

**Figure 4:**
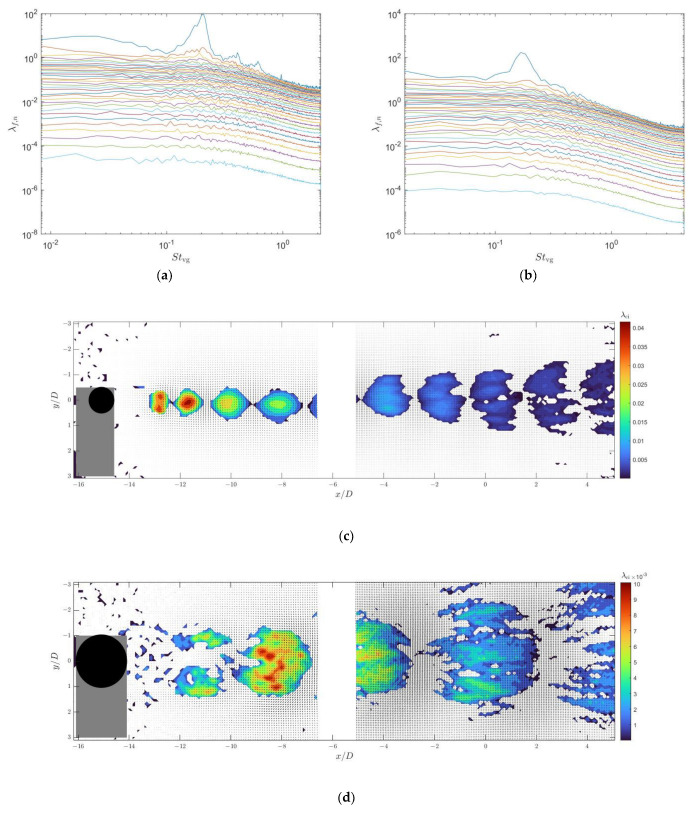
SPOD energy spectra of the wake flows of the type C1 and type C2 cylindrical vortex generators and the real part of the velocity fields and swirling strength corresponding to the peak modes ((**a**) energy spectrum of the type C1 cylindrical vortex generator wake flow; (**b**) energy spectrum of the type C2 cylindrical vortex generator wake flow; (**c**) real part of the velocity field distribution of the first mode at $St_{\mathrm{vg}}=0.207$ in the type C1 vortex generator wake flow; (**d**) real part of the velocity field of the first mode at $St_{\mathrm{vg}}=0.165$ in the type C2 vortex generator wake flow). The gray areas represent shadows caused by model obstruction and the white area between the two regions is the gap between the fields of view of the two cameras.

To demonstrate the complete modal characteristics of the wake flow field of the vortex generator, we performed a joint SPOD analysis on the velocity distribution of the vortex generator wake flow field captured by both upstream and downstream cameras. The analysis utilized 4800 flow field snapshots from each camera, with a segment length of 512 and an overlap rate of 75%. Figure 4a,b displays the SPOD energy spectrum and the highest-energy mode of the wake flow field when the cylinder is placed alone in a uniform incoming flow. The SPOD energy spectrum contains multiple curves, each representing the energy of the first-order mode, second-order mode, and so on, for different frequency components. Here, we plotted the energy of all 34 modes in the energy spectrum.

Furthermore, SPOD theory indicates that at a certain frequency, if the largest or several $\lambda_{f,n}$ values are significantly larger than the others, one or several physical coherent structures exist at that frequency, as these fluctuation modes absorb most of the turbulent kinetic energy at that frequency. The spatial distribution of the velocity field of the modes obtained from SPOD is a complex field, with a phase difference of π/2 between its real and imaginary parts. Therefore, in Figure 4 and subsequent modal velocity spatial distribution plots, displaying only the real part of each SPOD mode is sufficient to illustrate the contained spatial distribution information. In the spatial distribution plots, we use a contour plot of swirling strength $\lambda_{\mathrm{ci}}$ to identify vortices in the flow field. Swirling strength is defined as the imaginary part of the complex eigenvalues of the velocity gradient tensor (Adrian [22]).

From Figure 4a,b, it can also be found that the highest-energy mode $St_{\mathrm{vg}}$ for the type C1 cylinder wake flow is 0.207, while that for the type C2 cylinder wake flow is 0.165. The spatial modes corresponding to the highest-energy modes in the type C1 and C2 cylinder wake flows, as depicted in Figure 4c,d, are composed of periodically shed vortices forming a vortex street. Within these vortex streets, adjacent vortices have opposite rotational directions. The spatial period of the type C1 cylinder wake vortex street is approximately 4.0D, while that of the type C2 cylinder wake vortex street is approximately 8.0D.

To analyze the flow field modal characteristics related to the forces experienced by the sensor, we performed a separate SPOD of the velocity distribution around the sensor captured by the downstream camera. The number of snapshots, segment length, and overlap rate used in this decomposition were the same as those used in the analysis of the vortex generator wake.

**Figure 5:**
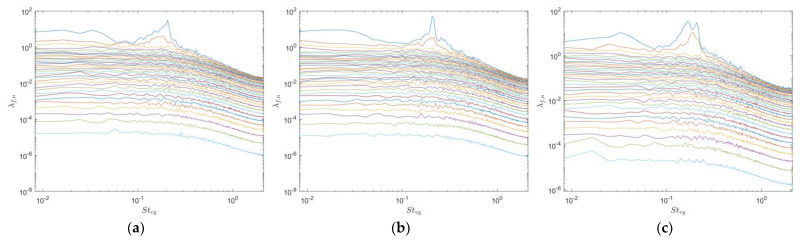

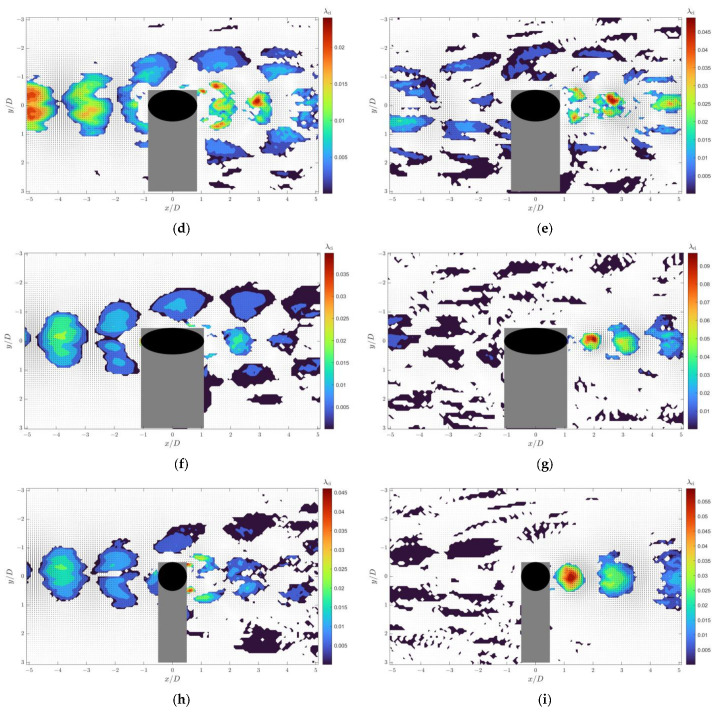
SPOD energy spectra of the flow fields around the sensors in the wake of a type C1 cylindrical vortex generator and the corresponding velocity fields and swirling strength at peak modes (**a**) energy spectrum of the upstream and downstream flow fields in the saddle plane of the seal whisker sensor; (**b**) energy spectrum of the upstream and downstream flow fields in the nodal plane of the seal whisker sensor; (**c**) energy spectrum of the upstream and downstream flow fields of the cylindrical sensor; (**d**) real part of the velocity field of the first mode at $St_{\mathrm{vg}}=0.207$ in the upstream and downstream flow fields in the saddle plane of the seal whisker sensor; (**e**) real part of the velocity field of the second mode at $St_{\mathrm{vg}}=0.182$ in the upstream and downstream flow fields in the saddle plane of the seal whisker sensor; (**f**) real part of the velocity field of the first mode at $St_{\mathrm{vg}}=0.207$ in the upstream and downstream flow fields in the nodal plane of the seal whisker sensor; (**g**) real part of the velocity field of the second mode at $St_{\mathrm{vg}}=0.215$ in the upstream and downstream flow fields in the nodal plane of the seal whisker sensor; (**h**) real part of the velocity field of the first mode at $St_{\mathrm{vg}}=0.207$ in the upstream and downstream flow fields of the cylindrical sensor; (**i**) real part of the velocity field of the first mode at $St_{\mathrm{vg}}=0.165$ in the upstream and downstream flow fields of the cylindrical sensor). The gray areas represent shadows caused by model obstruction and the white area between the two regions is the gap between the fields of view of the two cameras.

When the type C1 cylindrical vortex generator is present upstream, the flow field around the cylindrical sensor exhibits two first-order energy peak modes with similar energies and Strouhal numbers of 0.207 and 0.165, respectively (Figure 5c). The $St_{\mathrm{vg}}=0.207$ first-order mode corresponds to the vortex shedding from the upstream vortex generator. The shedding frequency and spatial distribution of the vortex street in the upstream region of the cylindrical sensor are unaffected, but the vortices dispersed upon encountering the cylindrical sensor due to obstruction (Figure 5g). The $St_{\mathrm{vg}}=0.165$ first-order mode corresponds to secondary vortex shedding downstream of the cylindrical sensor, with a frequency different from that of the vortex generator (Figure 5h).

In the flow field around the seal whisker sensor in the saddle plane, there is one first-order energy peak mode and one second-order energy peak mode, with Strouhal numbers of 0.207 and 0.182, respectively. The energy of the $St_{\mathrm{vg}}=0.207$ mode is approximately eight times that of the $St_{\mathrm{vg}}=0.182$ mode (Figure 5a). The $St_{\mathrm{vg}}=0.207$ first-order mode represents the vortex street downstream of the vortex generator, which remains unaffected in the upstream region of the seal whisker sensor but disperses upon reaching the sensor (Figure 5d). The $St_{\mathrm{vg}}=0.182$ second-order mode corresponds to secondary vortex shedding downstream of the seal whisker sensor, with a frequency different from that of the vortex generator (Figure 5e).

In the flow field around the seal whisker sensor in the node plane, there is also one first-order energy peak mode and one second-order energy peak mode, with Strouhal numbers of 0.207 and 0.215, respectively. The energies of these two peak modes are approximately equal, with the energy of the first-order mode being about fifteen times that of the second-order mode (Figure 5b). The $St_{\mathrm{vg}}=0.207$ first-order mode represents the vortex street downstream of the vortex generator, which remains unaffected in the upstream region of the seal whisker sensor but disperses upon reaching the sensor (Figure 5e). The $St_{\mathrm{vg}}=0.207$ second-order mode corresponds to secondary vortex shedding downstream of the seal whisker sensor, with a frequency similar to that of the vortex generator (Figure 5f).

**Figure 6:**
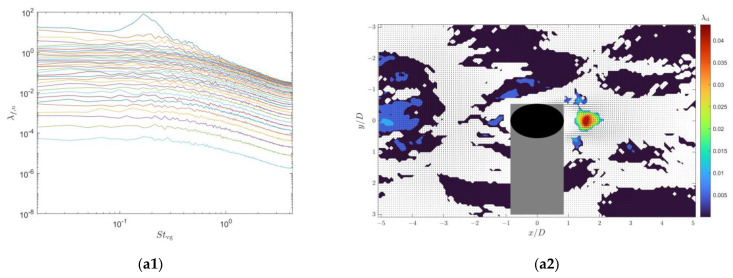

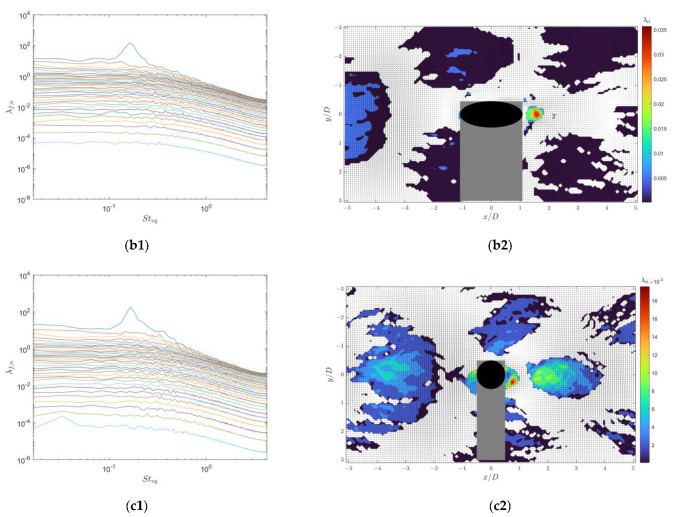
SPOD energy spectra of the flow fields around the sensors in the wake of the type C2 cylindrical vortex generator and the corresponding velocity fields and swirling strength at peak modes. The left three subfigures (**a1**,**b1**,**c1**) show the SPOD energy spectra of the flow fields in the saddle plane of the seal whisker, the nodal plane of the seal whisker, and the cross-section of the cylindrical sensor, respectively. The right three subfigures (**a2**,**b2**,**c2**) show the velocity fields and swirling strength corresponding to the modes with the highest SPOD energy. The gray areas represent shadows caused by model obstruction and the white area between the two regions is the gap between the fields of view of the two cameras.

When the type C2 cylindrical vortex generator is present upstream, the energy spectra of the upstream and downstream flow fields for both the cylindrical sensor and the two cross-sections of the seal whisker sensor each exhibit a single peak. This peak corresponds to a first-order mode with a Strouhal number of 0.165, which is the vortex shedding frequency of the upstream vortex generator (Figure 6(a1,b1,c1)). The spatial modes are dominated by the vortex shedding from the upstream vortex generator. In the upstream region of the cylindrical sensor, the shedding frequency and spatial distribution of the vortex street in the incoming flow remain unaffected by the sensor. However, as the vortices reach the cylindrical sensor, they split due to obstruction and generate secondary vortex shedding at the same frequency in a smaller region directly downstream of the sensor (Figure 6(a2,b2,c2)).

## 4. Discussion

### 4.1. Vortex-Induced Lift and SPOD Modes

By correlating the lift signals received by the sensors with the frequencies of the peak SPOD modes, it can be observed that the two lift peaks ($St_{\mathrm{vg}}=0.187$ and $St_{\mathrm{vg}}=0.149$) of the cylindrical sensor in the wake of the type C1 cylindrical vortex generator correspond to the two first-order peak modes ($St_{\mathrm{vg}}=0.207$ and $St_{\mathrm{vg}}=0.165$) in the SPOD analysis. The relative errors between the lift peak frequencies and the corresponding SPOD mode peak frequencies are both approximately −10%. Although the strengths of these two modes are similar in the SPOD energy spectrum, the lift signal component corresponding to the vortex shedding from the vortex generator is much larger than that induced by the secondary vortex shedding.

For the seal whisker sensor in the wake of the type C1 cylindrical vortex generator, there are two lift peaks at $St_{\mathrm{vg}}=0.186$ and $St_{\mathrm{vg}}=0.145$. In the saddle plane, there is a first-order peak at $St_{\mathrm{vg}}=0.207$ and a second-order peak at $St_{\mathrm{vg}}=0.165$, while in the nodal plane, there is only a first-order peak at $St_{\mathrm{vg}}=0.207$. The stronger lift peak at $St_{\mathrm{vg}}=0.186$ corresponds to the vortex shedding mode ($St_{\mathrm{vg}}=0.207$) in both cross-sections, with a relative error of approximately −10%. When an upstream vortex generator is introduced, placing the seal whisker sensor within its vortical wake, the transverse back-and-forth movement of fluid in the vortices subjects the whisker to an oscillating lift. This results in a larger measured lift amplitude compared to conditions without the vortex generator in the incoming flow.

When placed in the wake of the type C2 cylindrical vortex generator, the lift peak of the cylindrical sensor at $St_{\mathrm{vg}}=0.151$ and the lift peak of the seal whisker sensor at $St_{\mathrm{vg}}=0.161$ both correspond to the highest-energy SPOD mode at $St_{\mathrm{vg}}=0.165$, with a relative error of approximately −8%. These results indicate that the primary source of the lift signals for both the cylindrical and seal whisker sensors is the vortices in the incoming flow. Secondary vortex shedding downstream, if differing in frequency from the incoming flow vortices, can interfere with the sensor’s measurements. In the wake of the type C1 cylindrical vortex generator, the secondary vortex shedding from the seal whisker sensor contains fewer energetic frequency components compared to that from the cylindrical sensor, resulting in less interference and better noise reduction performance.

### 4.2. Secondary Vortex Shedding and Array Interference

Secondary vortex shedding downstream of a sensor not only exerts forces on the sensor but can also interfere with downstream sensors in an array configuration. SPOD results reveal that in the wake of the type C2 cylindrical vortex generator, the primary modes downstream of both the cylindrical and seal whisker sensors are at the same frequency as the vortex generation source, with weak secondary vortex shedding. Therefore, it is less likely to cause significant interference with the lift measurements of downstream sensors. In the wake of the type C1 cylindrical vortex generator, the cylindrical sensor experiences strong secondary vortex shedding downstream, which can interfere with the lift signals of downstream sensors. For the seal whisker sensor, the secondary vortex shedding mode in the nodal plane occurs at the same frequency as the upstream vortex shedding, whereas in the saddle plane, weak secondary vortex shedding modes appear at different frequencies. The unique geometry of the seal whisker enables it not only to suppress vortex shedding in the absence of a vortex generator, but also to effectively dampen secondary vortex shedding in flows containing vortices—a capability that cylindrical sensors lack. As a result, the seal whisker sensor causes less disturbance to downstream sensors in an array configuration, making it a more favorable choice for sensor array applications.

### 4.3. Lift Reception Capability of Seal Whisker-Shaped Cylinder

Comparing the lift forces experienced by the cylindrical and seal whisker sensors in the wake of the type C2 cylindrical vortex generator, it is found that the spectral peak of the lift force experienced by the seal whisker sensor is approximately three times that of the cylindrical sensor. In the wake of the type C1 cylindrical vortex generator, the differences in the spectral peaks of the lift forces are not as significant. SPOD analysis shows that in the first-order mode of the type C1 cylindrical vortex generator wake, the spacing between adjacent vortices is 2.0D, and the streamwise size of a single vortex is less than 2.0D. The streamwise size of the cylindrical sensor is 1.0D, and that of the seal whisker sensor is 2.0D. Both sensors can receive the lift force induced by nearly a complete vortex, resulting in similar lift forces. In the first-order mode of the type C2 cylindrical vortex generator wake, the spatial period of the vortex street is approximately 8.0D, and the streamwise size of a single vortex is less than 4.0D. The seal whisker sensor, being larger in the streamwise direction than the cylindrical sensor, can receive the lift force induced by a larger area of the same vortex, resulting in a higher lift force compared to the cylindrical sensor.

When the incoming flow contains larger-scale vortices, the characteristic shape of the seal whisker enables it to measure a larger peak lift amplitude than the cylindrical sensor, demonstrating superior signal capture capabilities. The experiments by Zheng et al. [4] also measured and compared the vibration signals of a cylinder and a seal whisker in both uniform incoming flow and wake flows. In their experiments, when no vortex generator is present, the vibration signal of the cylinder was stronger than that of the seal whisker, a finding consistent with our experiments. However, in incoming flows with a vortex generator, their results indicated that the vibration signal of the cylinder was also stronger than that of the seal whisker. This contrasts with our findings, where the peak lift measured by the seal whisker sensor was slightly higher than that of the cylindrical sensor in the type C1 cylindrical vortex generator wake, and significantly higher in the type C2 cylindrical vortex generator wake. This discrepancy may be attributed to differences in the experimental fixation method and the physical quantity measured. In our experiments, the sensor body was completely fixed, and we measured the lift force exerted on it. In contrast, their sensor was mounted on a hinge, and they measured the rotation of the whisker base hinge. The strength of this rotational signal is influenced not only by the magnitude of the fluid lift force experienced by the structure but also by its dynamic parameters, such as the moment of inertia of both the whisker and the cylinder.

## 5. Conclusions

We conducted experiments in a water channel to investigate the performance of seal whisker and cylindrical sensors in the uniform incoming flow with no vortex generator present, as well as in the wake of type C1 and type C2 cylindrical vortex generators. The lift forces experienced by the sensors were measured, and the velocity fields were obtained using the PIV method.

FFT spectral analysis of the lift signals revealed that when no vortex generator is present, the lift amplitude of the seal whisker sensor is weaker than that of the cylindrical sensor. In the wake of the vortex generators, the peak frequencies of the lift spectra for both sensors are close to the vortex shedding frequencies of the upstream vortex generators. In the case of no vortex generator and in the wake of the type C1 cylindrical vortex generator, the seal whisker sensor exhibits less noise in the lift signal compared to the cylindrical sensor, demonstrating better noise reduction performance. In the wake of the type C2 cylindrical vortex generator, the seal whisker sensor receives a stronger lift signal than the cylinder sensor, indicating better signal capture capability.

An analysis of the PIV-measured velocity fields, along with the SPOD modal characteristics and the lift forces, revealed that the primary source of the lift signals for both the cylindrical and seal whisker sensors is the vortices in the incoming flow. Under all experimental conditions, the lift signal detected by the seal whisker sensor is clearer compared to that from the cylinder sensor. SPOD modal analysis further indicates that secondary vortex shedding downstream of the cylinder sensor is more pronounced, with a frequency distinct from that of the incoming vortex street, which contributes to the higher noise levels in the lift signal of the cylinder sensor. Additionally, in comparison to the type C1 cylindrical vortex generator, the lift amplitude detected by the seal whisker sensor is significantly enhanced in the wake of the type C2 vortex generator, whereas the lift amplitude from the cylinder sensor shows a slight decrease. This variation is partially attributed to the difference in the scale of the wake vortices.

In summary, compared to the cylindrical sensor, the seal whisker sensor effectively suppresses secondary vortex shedding in incoming flows containing vortex generators. This characteristic is advantageous as it minimizes interference during the detection of wake flows behind obstacles and reduces inter-whisker interference when sensors are arranged in an upstream–downstream array. However, due to limitations in the current experimental setup, this study did not account for the curvature and variable cross-sections inherent in actual seal whisker morphology, nor the potential effects of varying angles of attack on the measurement outcomes. Future advancements in our experimental capabilities will be essential to test more complex geometric features such as characteristic curvature and tapering and to explore their performance under a wider range of dynamic conditions. These investigations will then extend to characterizing the detection performance and flow field dynamics within sensor arrays, ultimately providing deeper insights for optimizing bio-inspired sensing systems.

## Figures and Tables

**Table 1:** Peak data from the lift FFT spectra measured by the cylindrical sensor and the seal whisker sensor under three different scenarios.

Sensor Type	Vortex Generator Type	Peak 1		Peak 2	
		St *	${\hat{F}}_{L}$	St	${\hat{F}}_{L}$
Seal Whisker	No	- **	-	-	-
	C1	0.186	0.137	0.145	0.043
	C2	0.161	0.214	-	-
Circular Cylinder	No	0.188	0.006	-	-
	C1	0.187	0.103	0.149	0.065
	C2	0.151	0.077	-	-

* When a vortex generator is present, St represents $St_{\mathrm{vg}}$; in the absence of a vortex generator, St denotes $St_{s}$. ** indicates no detectable peak.

## Data Availability

Data are contained within the article.

## Abbreviations

The following abbreviations are used in this manuscript:

SPOD	Spectral proper orthogonal decomposition
PIV	Particle image velocimetry
VIV	Vortex-induced vibrations
POD	Proper orthogonal decomposition
DMD	Dynamic mode decomposition
FFT	Fast Fourier transform
